# Systemic exposure following intravitreal administration of therapeutic agents: an integrated pharmacokinetic approach. 2. THR-687

**DOI:** 10.1007/s10928-021-09774-9

**Published:** 2021-07-23

**Authors:** Marc Vanhove, Jean-Marc Wagner, Bernard Noppen, Bart Jonckx, Elke Vermassen, Alan W. Stitt

**Affiliations:** 1 Oxurion N.V, Gaston Geenslaan 1, 3001 leuven, Belgium; 2 Haute École de la Province de Liège, Avenue Montesquieu 6, 4101 Seraing, Belgium; 3 Centre for Experimental Medicine, Queen’s University Belfast, Belfast, Northern Ireland, UK

**Keywords:** Intravitreal administration, Systemic exposure, Integrated pharmacokinetics

## Abstract

Intravitreal (IVT) injection remains the preferred administration route of pharmacological agents intended for the treatment of back of the eye diseases such as diabetic macular edema (DME) and neovascular age-related macular degeneration (nvAMD). The procedure enables drugs to be delivered locally at high concentrations whilst limiting whole body exposure and associated risk of systemic adverse events. Nevertheless, intravitreally-delivered drugs do enter the general circulation and achieving an accurate understanding of systemic exposure is pivotal for the evaluation and development of drugs administered in the eye. We report here the full pharmacokinetic properties of THR-687, a pan RGD integrin antagonist currently in clinical development for the treatment of DME, in both rabbit and minipig. Pharmacokinetic characterization included description of vitreal elimination, of systemic pharmacokinetics, and of systemic exposure following IVT administration. For the latter, we present a novel pharmacokinetic model that assumes clear partition between the vitreous humor compartment itself where the drug is administered and the central systemic compartment. We also propose an analytical solution to the system of differential equations that represent the pharmacokinetic model, thereby allowing data analysis with standard nonlinear regression analysis. The model accurately describes circulating levels of THR-687 following IVT administration in relevant animal models, and we suggest that this approach is relevant to a range of drugs and analysis of subsequent systemic exposure.

## Introduction

Diabetic macular edema (DME) and neovascular age-related macular degeneration (nvAMD) are the leading causes of visual impairment in developed countries [1–3]. Among the range of treatment options, intravitreal (IVT) administration, which allows to achieve high local concentrations while limiting systemic exposure, is by far the preferred administration route of pharmacological agents used or investigated for the treatment of these retinal disorders [4].

In principle, drugs injected in the vitreous could be cleared from the eye either via local metabolic elimination or physical elimination to the blood circulation, the latter occurring preferentially though the anterior or posterior routes depending on the nature and physico-chemical properties of the drug [5]. Recent data also suggest the existence of both an ocular glymphatic route, which would allow drugs to be cleared to the cerebrospinal fluid via the paravenous space, and of an ocular lymphatic drainage system [6–8]. However, although some hydrolytic, e.g. esterase and peptidase, activity exists in the eye vitreous [9] and proved sufficient to allow conversion of ester pro-drugs of acyclovir and ganciclovir [10], most drugs are eliminated via the blood stream and subsequent systemic elimination [5]. Intravitreally-administered anti-vascular endothelial growth factor (VEGF) agents, which usage has revolutionized the treatment of DME and nvAMD [11, 12], are no exception, and drainage into the systemic circulation results into drug concentrations sufficient to significantly affect circulating VEGF levels [13–15]. It is also relevant to note that despite being very effective, anti-VEGF agents require multiple and regular injections, which potentiate the risk of systemic toxicity associated with suppression of circulating VEGF.

Pharmacokinetic models which describe or predict circulating drug levels following IVT administration are essential to evaluate the degree of systemic exposure and potential toxicity. In an accompanying paper (Vanhove et al., this issue of J. Pharmacokinet. Pharmacodyn. 10.1007/s10928-021-09773-w), we attempted to model circulating levels following IVT administration of THR-149, a plasma kallikrein bicyclic peptide inhibitor currently being developed by Oxurion N.V. (Belgium) for the treatment of DME patients who are sub-optimal responders to anti-VEGFs. In this paper, we report the pharmacokinetic properties in rabbit and minipig of THR-687, a pan RGD integrin small molecule antagonist [16] also being developed by Oxurion N.V. for the treatment of DME (source: www.clinicaltrials.gov). Circulating THR-687 levels following injection in the eye were interpreted based on a novel multi-compartmental pharmacokinetic model which shares similarities with the one used to model circulating levels of THR-149 but specifically applies to drugs which intravenous pharmacokinetics are described by a standard bi-compartmental model. We propose that these models are broadly applicable to describe plasma levels of drugs delivered into the eye. We also report here, like in our accompanying paper, the analytical solution to the system of linear differential equations that represents the model, which allows data treatment by standard nonlinear regression analysis and should, therefore, facilitate the utilization of the model by a broader range of scientists.

## Methods

### Nonlinear regression analyses.

Nonlinear regression analyses were performed using the GraphPad Prism software ver. 5.02 (GraphPad Software Inc., La Jolla, CA) applying, as indicated, either equal weighting (i.e. performing minimization based on absolute distances squared) or proportional weighting (i.e. performing minimization based on relative distances squared). Specifically for the analysis of plasma levels measured following IVT administration with Eq. 19, and in order to compute unique values for either the parameter k_2_ or the parameters k_2_ and V_D,syst_, the data sets were analyzed “globally”, i.e. considering that the data obtained for the different doses represent a unique set of data, and with k_2_ or k_2_ and V_D,syst_ used as a “shared” parameter(s) (see also our accompanying paper). Precision on the fitted parameters was expressed as 95% confidence intervals (CI_95%_).

### Intravitreal pharmacokinetics

Intravitreal pharmacokinetics following bilateral administration of 3 mg per eye of THR-687 in New-Zealand White (NZW) rabbits was reported previously [16]. Experimental data (THR-687 concentration *vs*. time) were analyzed based on a mono-compartmental model (Eq. 1) applying a proportional weighting, as described in our accompanying paper. Vitreous humor (VH) half-life and vitreal clearance (CL_VH_) were calculated using Eq. 2 and 3.

1 $${[{\text{THR}}-687]}_{VH}=\frac{D}{{V}_{D,VH}}\cdot {e}^{-{k}_{1}\cdot t}$$

2 $${t}_{1/2}=\frac{ln(2)}{{k}_{1}}$$

3 $${CL}_{VH}={k}_{1}\cdot {V}_{D,VH}$$

### Intravenous pharmacokinetics

Studies were performed at Charles River Laboratories (Montreal, Canada). NZW rabbits (3 males, 3 females, average weight 3.4 kg) and Gottingen minipigs (3 males, 3 females, average weight 14 kg) received on day 1 a dose of 25 mg (rabbits) or 50 mg (minipigs) of THR-687 as an intravenous bolus. As part of the study, the animals received the same 25 or 50 mg dose on days 15, 29, 43, 57, and 71. Blood samples for doses administered on days 1 and 71 were collected 0.5, 1, 2, 4, and 8 h after drug administration. Samples were processed to plasma and analyzed using a validated liquid chromatography with tandem mass spectrometry (LC–MS/MS) method. Data collected for animals dosed at day 1 and at day 71 were essentially identical and were thus pooled to increase sample size.

**Fig. 1:**
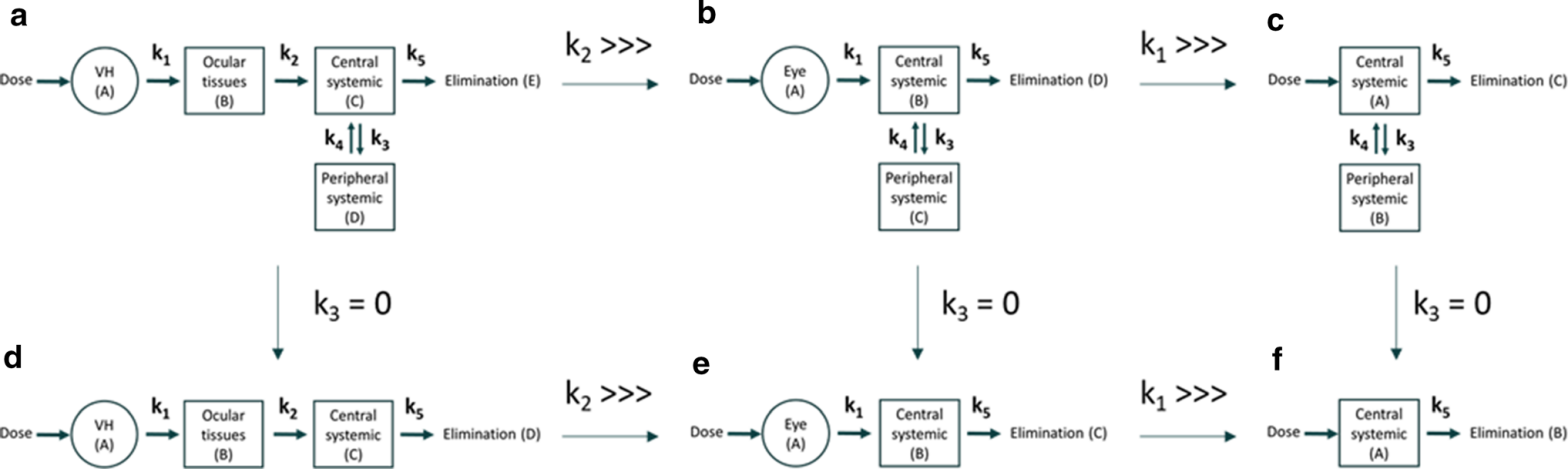
Compartmental pharmacokinetic models. **a** Intravitreal administration assuming the presence of an ocular tissues compartment and bi-compartmental systemic distribution. **b** Intravitreal administration assuming bi-compartmental systemic distribution. **c** Intravenous administration assuming bi-compartmental systemic distribution. **d** Intravitreal administration assuming the presence of an ocular tissues compartment and mono-compartmental systemic distribution. **e** Intravitreal administration assuming mono-compartmental systemic distribution. **f** Intravenous administration assuming mono-compartmental systemic distribution

Data (THR-687 plasma concentration *vs*. time) were analyzed based on a bi-compartmental model (model (c) in Fig. 1) using Eq. 4 (with D the dose, V_D,syst_ the volume of distribution of the central systemic compartment, and k_3_, k_4_ & k_5_ the individual rate constants characterizing the model) applying equal weighting for the determination of V_D,syst_ and—in order to provide enough weight to the low concentration values—proportional weighting for the determination of k_3_, k_4_ & k_5_. Of note, Eq. 4 is identical to the equations historically described for this pharmacokinetic model [17] but re-arranged is such a way that data fitting directly provides the value of the individual rate constants without the need for additional calculations. The systemic clearance (CL_syst_) and terminal half-life (t_1/2-term_) were obtained from Eq. 5 & 6, respectively.

4 $${[{\text{THR}}-687]}_{syst}=\frac{D}{{V}_{D,syst}\cdot \left(\beta -\alpha \right)}\cdot \left\{{\left({k}_{4}-\alpha \right)\cdot e}^{-\alpha \cdot t}+{\left(\beta -{k}_{4}\right)\cdot e}^{-\beta \cdot t}\right\}$$

with

$$\alpha =\frac{1}{2}\cdot \left\{{k}_{3}+{k}_{4}+{k}_{5}+\sqrt{{\left({k}_{3}+{k}_{4}+{k}_{5}\right)}^{2}-4\cdot {k}_{4}\cdot {k}_{5}}\right\}$$

$$\beta =\frac{1}{2}\cdot \left\{{k}_{3}+{k}_{4}+{k}_{5}-\sqrt{{\left({k}_{3}+{k}_{4}+{k}_{5}\right)}^{2}-4\cdot {k}_{4}\cdot {k}_{5}}\right\}=\frac{{k}_{4}\cdot {k}_{5}}{\alpha }$$

5 $${CL}_{syst}={k}_{5}\cdot {V}_{D,syst}$$

6 $${t}_{1/2-term}=\frac{ln(2)}{\beta }$$

### Plasma levels following intravitreal administration

Studies were performed at Charles River Laboratories (Montreal, Canada). NZW rabbits (average weight 3.4 kg) and Gottingen minipigs (average weight 14 kg) received on day 1 a bilateral 50-µL IVT injection of THR-687 at doses of 0.2, 1 or 5 mg per eye (rabbits, 3 males and 3 females for the 0.2 and 1 mg doses, 6 males and 6 females for the 5 mg dose) or 1, 3 or 10 mg per eye (minipigs, 3 males and 3 females for the 1 and 3 mg doses, 6 males and 6 females for the 10 mg dose). Like for the intravenous pharmacokinetic study, the animals also received an identical dose on days 15, 29, 43, 57, and 71. Blood samples for doses administered on days 1 and 71 were collected 0, 2, 8, 16, 24, 32, and 48 h after drug administration, samples were processed to plasma, and circulating THR-687 levels were determined as described above. Data collected for animals dosed at day 1 and at day 71 were essentially identical and were pooled to increase sample size. Data were analyzed based on pharmacokinetic models (a) or (b) from Fig. 1, i.e. using Eq. 12 or 19 with equal weighting.

Model (b) can be described by the system of linear differential equations represented below (Eq. syst. 1, with A, B, and C representing the VH, the central systemic and the peripheral systemic compartments, respectively, and D representing the eliminated drug), the solution (integration) of which provides the variation of the drug concentration in each compartment as a function of time (see Appendix section). We also refer here the reader to our accompanying paper for a detailed description of a method based on matrices operations that can be used to solve such systems of differential equations.

$$ \begin{aligned} dA/dt & = - k_{1} \cdot A\quad {\text{Eq}}.{\text{syst}}{.1} \\ dB/dt & = k_{1} \cdot A - (k_{3} + k_{5} ) \cdot B + k_{4} \cdot C \\ dC/dt & = k_{3} \cdot B - k_{4} \cdot C \\ dD/dt & = k_{5} \cdot B \\ \end{aligned} $$

For readers who might have a use for these equations, and because these have never been reported to our knowledge, we provide here the full solution of Eq. syst. 1 (Eq. 7–10, with α and β as in Eq. 4—see also the Appendix section). This also allows to verify the validity of the proposed solutions, since it can be shown that A_t_ + B_t_ + C_t_ + D_t_ = A_0_ at all time points.

7 $${A}_{t}={A}_{0}\cdot {e}^{- {k}_{1}\cdot t}$$

8 $${B}_{t}={A}_{0}\cdot \left\{\frac{{k}_{1}\cdot \left({k}_{4}-{k}_{1}\right)}{\left({k}_{1}-\alpha \right)\cdot \left({k}_{1}-\beta \right)}\cdot {e}^{- {k}_{1}\cdot t}+\frac{{\alpha \cdot k}_{1}\cdot \left({k}_{5}-\beta \right)}{{k}_{5}\cdot \left(\alpha -\beta \right)\cdot \left({k}_{1}-\alpha \right)}\cdot {e}^{- \alpha \cdot t}+\frac{{\beta \cdot k}_{1}\cdot \left(\alpha -{k}_{5}\right)}{{k}_{5}\cdot \left(\alpha -\beta \right)\cdot \left({k}_{1}-\beta \right)}\cdot {e}^{- \beta \cdot t}\right\}$$

9 $${C}_{t}={A}_{0}\cdot \left\{\frac{{k}_{1}\cdot {k}_{3}}{\left({k}_{1}-\alpha \right)\cdot \left({k}_{1}-\beta \right)}\cdot {e}^{- {k}_{1}\cdot t}+\frac{{k}_{1}\cdot \left({k}_{5}-\alpha \right)\cdot \left({k}_{5}-\beta \right)}{{k}_{5}\cdot \left(\alpha -\beta \right)\cdot \left({k}_{1}-\alpha \right)}\cdot {e}^{- \alpha \cdot t}+\frac{{k}_{1}\cdot \left({k}_{5}-\alpha \right)\cdot \left(\beta -{k}_{5}\right)}{{k}_{5}\cdot \left(\alpha -\beta \right)\cdot \left({k}_{1}-\beta \right)}\cdot {e}^{- \beta \cdot t}\right\}$$

10 $${D}_{t}={A}_{0}\cdot \left\{\frac{{k}_{5}\cdot \left({k}_{1}-{k}_{4}\right)}{\left({k}_{1}-\alpha \right)\cdot \left({k}_{1}-\beta \right)}\cdot {e}^{- {k}_{1}\cdot t}+\frac{{k}_{1}\cdot \left[{k}_{3}\cdot \beta -\left({\beta -k}_{5}\right)\cdot \left({k}_{1}-{k}_{4}\right)\right]}{\left(\beta -\alpha \right)\left({k}_{1}-\alpha \right)\cdot \left({k}_{1}-\beta \right)}\cdot {e}^{- \alpha \cdot t}+\frac{{k}_{1}\cdot \left[\left({\alpha -k}_{5}\right)\cdot \left({k}_{1}-{k}_{4}\right)-{k}_{3}\cdot \alpha \right]}{\left(\beta -\alpha \right)\left({k}_{1}-\alpha \right)\cdot \left({k}_{1}-\beta \right)}\cdot {e}^{- \beta \cdot t}+\frac{{k}_{5}\cdot \left({k}_{1}-{k}_{4}\right)-{k}_{1}\cdot \left({k}_{1}-{k}_{3}-{k}_{4}\right)}{\left({k}_{1}-\alpha \right)\cdot \left({\beta -k}_{1}\right)}\right\}$$

For the VH and central systemic compartments, i.e. the compartments that can be experimentally sampled, the mathematical expressions that represent the variation of the drug concentration over time are thus in the form of Eq. 11 & 12 (with Eq. 11 being, as expected, identical to Eq. 1). Of note, the term D in Eq. 12 represents the total dose, i.e. twice the dose administered in each eye in case of bilateral administration.

11 $${A}_{t}=\frac{D}{{V}_{D,VH}}\cdot {e}^{- {k}_{1}\cdot }t$$

12 $${B}_{t}=\frac{D}{{V}_{D,syst}}\cdot \left\{\frac{{k}_{1}\cdot \left({k}_{4}-{k}_{1}\right)}{\left({k}_{1}-\alpha \right)\cdot \left({k}_{1}-\beta \right)}\cdot {e}^{- {k}_{1}\cdot t}+\frac{{\alpha \cdot k}_{1}\cdot \left({k}_{5}-\beta \right)}{{k}_{5}\cdot \left(\alpha -\beta \right)\cdot \left({k}_{1}-\alpha \right)}\cdot {e}^{- \alpha \cdot t}+\frac{{\beta \cdot k}_{1}\cdot \left(\alpha -{k}_{5}\right)}{{k}_{5}\cdot \left(\alpha -\beta \right)\cdot \left({k}_{1}-\beta \right)}\cdot {e}^{- \beta \cdot t}\right\}$$

Similarly, model (a) in Fig. 1 can be described by Eq. syst. 2, with A, B, C, and D representing the VH, the ocular tissues, the central systemic and the peripheral systemic compartments, respectively, and E representing the eliminated drug. The solutions of Eq. syst. 2 are provided (Eq. 13–17, see also the Appendix section), and the mathematical expressions that represent the variation of the drug concentration over time in the VH and central systemic compartments are thus in the form of Eq. 18 & 19 (again with Eq. 18 being identical to Eq. 1). As for Eq. 12, the term D in Eq. 19 represents the total dose, i.e. twice the dose administered in each eye in case of bilateral administration.

$$ \begin{aligned} dA/dt & = - k_{1} \cdot A\quad {\text{Eq}}.{\text{syst}}{.2} \\ dB/dt & = k_{1} \cdot A - k_{2} \cdot B \\ dC/dt & = k_{2} \cdot B - (k_{3} + k_{5} ) \cdot C + k_{4} \cdot D \\ dD/dt & = k_{3} \cdot C - k_{4} \cdot D \\ dE/dt & = k_{5} \cdot C \\ \end{aligned} $$

13 $${A}_{t}={A}_{0}\cdot {e}^{- {k}_{1}\cdot t}$$

14 $$B_{t} = \frac{{A_{0} \cdot k_{1} }}{{\left( {k_{1} - k_{2} } \right)}} \cdot \left( {e^{{ - k_{2} \cdot t}} - e^{{ - k_{1} \cdot t}} } \right)$$

15 $${C}_{t}=\frac{{A}_{0}\cdot {k}_{1}\cdot {k}_{2}}{\left({k}_{1}-{k}_{2}\right)\cdot M\cdot R\cdot N}\cdot \left\{\left({k}_{1}-{k}_{4}\right)\cdot R\cdot N\cdot {e}^{- {k}_{1}\cdot t}-\left({k}_{2}-{k}_{4}\right)\cdot M\cdot N\cdot {e}^{- {k}_{2}\cdot t}+\left[-\left({k}_{1}-{k}_{4}\right)\cdot R\cdot N+\left({k}_{2}-{k}_{4}\right)\cdot M\cdot N+U\right]\cdot {e}^{- \alpha \cdot t}-U\cdot {e}^{- \beta \cdot t}\right\}$$

16 $${D}_{t}=\frac{{A}_{0}\cdot {k}_{1}\cdot {k}_{2}\cdot {k}_{3}}{\left({k}_{1}-{k}_{2}\right)\cdot M\cdot R\cdot N}\cdot \left\{-R\cdot N\cdot {e}^{- {k}_{1}\cdot t}+M\cdot N\cdot {e}^{- {k}_{2}\cdot t}+\frac{\left({k}_{1}-{k}_{4}\right)\cdot R\cdot N-\left({k}_{2}-{k}_{4}\right)\cdot M\cdot N-U}{\alpha -{k}_{4}}\cdot {e}^{- \alpha \cdot t}+\left[R\cdot \left(\alpha -{k}_{1}\right)-M\cdot \left(\alpha -{k}_{2}\right)\right]\cdot {e}^{- \beta \cdot t}\right\}$$

17 $${E}_{t}=\frac{{A}_{0}\cdot {k}_{5}}{\left({k}_{1}-{k}_{2}\right)}\cdot \left\{-\frac{{k}_{2}\cdot \left({k}_{1}-{k}_{4}\right)}{M}\cdot {e}^{- {k}_{1}\cdot t}+\frac{{k}_{1}\cdot \left({k}_{2}-{k}_{4}\right)}{R}\cdot {e}^{- {k}_{2}\cdot t}-\frac{{k}_{1}\cdot {k}_{2}\cdot \left[-\left({k}_{1}-{k}_{4}\right)\cdot R\cdot N+\left({k}_{2}-{k}_{4}\right)\cdot M\cdot N+U\right]}{\alpha \cdot M\cdot R\cdot N}\cdot {e}^{- \alpha \cdot t}+\frac{{k}_{1}\cdot {k}_{2}\cdot U}{\beta \cdot M\cdot R\cdot N}\cdot {e}^{- \beta \cdot t}+\frac{{k}_{2}\cdot \left({k}_{1}-{k}_{4}\right)\cdot \alpha \cdot \beta \cdot R\cdot N-{k}_{1}\cdot \left({k}_{2}-{k}_{4}\right)\cdot \alpha \cdot \beta \cdot M\cdot N+{k}_{1}\cdot {k}_{2}\cdot \beta \cdot \left[-\left({k}_{1}-{k}_{4}\right)\cdot R\cdot N+\left({k}_{2}-{k}_{4}\right)\cdot M\cdot N+U\right]-{k}_{1}\cdot {k}_{2}\cdot \alpha \cdot U}{\alpha \cdot \beta \cdot M\cdot R\cdot N}\right\}$$

18 $${A}_{t}=\frac{D}{{V}_{D,VH}}\cdot {e}^{- {k}_{1}\cdot t}$$

19 $${C}_{t}=\frac{D\cdot {k}_{1}\cdot {k}_{2}}{{V}_{D,syst}\cdot \left({k}_{1}-{k}_{2}\right)\cdot M\cdot R\cdot N}\cdot \left\{\left({k}_{1}-{k}_{4}\right)\cdot R\cdot N\cdot {e}^{- {k}_{1}\cdot t}-\left({k}_{2}-{k}_{4}\right)\cdot M\cdot N\cdot {e}^{- {k}_{2}\cdot t}+\left[-\left({k}_{1}-{k}_{4}\right)\cdot R\cdot N+\left({k}_{2}-{k}_{4}\right)\cdot M\cdot N+U\right]\cdot {e}^{- \alpha \cdot t}-U\cdot {e}^{- \beta \cdot t}\right\}$$

With α and β as in Eq. 4 and:

$$ \begin{aligned} M & = k_{1}^{2} - k_{1} \cdot k_{3} - k_{1} \cdot k_{4} - k_{1} \cdot k_{5} + k_{4} \cdot k_{5} \\ R & = k_{2}^{2} - k_{2} \cdot k_{3} - k_{2} \cdot k_{4} - k_{2} \cdot k_{5} + k_{4} \cdot k_{5} \\ N & = \sqrt {\left( {k_{3} + k_{4} + k_{5} } \right)^{2} - 4 \cdot k_{4} \cdot k_{5} } \\ U & = \left( {\beta - k_{4} } \right) \cdot \left\{ {R \cdot \left( {\alpha - k_{1} } \right) - M \cdot \left( {\alpha - k_{2} } \right)} \right\} \\ \end{aligned} $$

### Binding to plasma proteins

Binding of THR-687 to rabbit, pig, and human plasma proteins was determined by equilibrium dialysis. Rabbit, pig, and human plasma samples were purchased from Bio-Connect (cat. GTX73225), Nodia (cat. IPG-N-500 ml-Na Citr), and Innovative Research Inc. (cat. 27,744), respectively. THR-687 was spiked in plasma at final concentrations of 10 and 100 µM and samples where dialyzed against phosphate buffered saline (PBS) until equilibrium using the Rapid Equilibrium Dialysis (RED) Device 8 K MWCO from ThermoFisher Scientific (cat. 90,006). Alternatively, the drug was spiked in the PBS compartment. THR-687 concentrations in both the PBS and plasma compartments were then determined by liquid chromatography–mass spectrometry (LC–MS). Chromatography separation was achieved on an Acquity UPLC coupled to a QDa instrument (Waters). Prior to analysis, the dialyzed plasma and PBS samples were diluted with 1 volume of PBS and 1 volume of control plasma, respectively, followed by a dilution with 3 volumes of 0.1% (v/v) formic acid in acetonitrile and clarified by centrifugation for 10 min at 13,000 rpm. All samples were then diluted in 10% (v/v) acetonitrile and 0.1% (v/v) formic acid, and 6 µL of the diluted samples were injected on a BEH C18 300A, 1.7 µm, 2.1 × 100 mm Acquity UPLC column (Waters, cat. 186,003,686) pre-equilibrated in 10% (v/v) acetonitrile and 0.1% (v/v) formic acid. The temperature of the column was maintained at 65 °C. Elution was performed by applying a 3-mL, 10 to 33% (v/v) acetonitrile linear gradient in 0.1% (v/v) formic acid at a flow rate of 600 µL/min. THR-687 was detected in positive polarity single ion recording mode via its [M + 2H]^2+^. The concentration of THR-687 in the samples was calculated by integration of the relevant peak and reference to a standard curve obtained by injection of a series of samples of known concentration. The percentage of THR-687 bound to plasma proteins was calculated by comparing drug levels in plasma and in the corresponding PBS compartment.

## Results

### Intravitreal pharmacokinetics

**Fig. 2:**
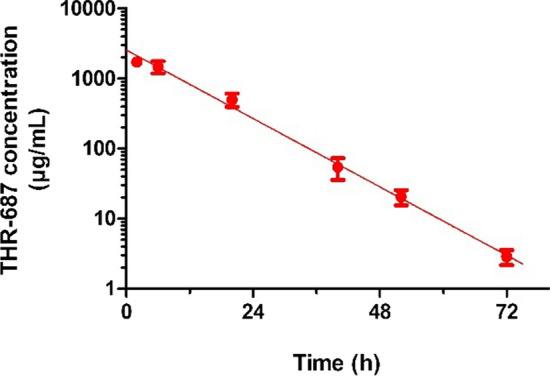
Pharmacokinetics in the rabbit VH following IVT administration of 3 mg of THR-687. Data are shown as mean ± SD. The solid line represents the best fit given by Eq. 1. THR-687 is eliminated from the rabbit VH with a half-life of 7.4 h [16]

Rabbits are the most commonly used animal species for the determination of intravitreal pharmacokinetics, and translation to human appears feasible despite some anatomical differences and differences in the properties of the vitreous humor [18]. Pharmacokinetic properties following IVT administration of THR-687 in rabbit were reported by Hu et al. [16] and are also shown in Fig. 2. THR-687 was found to be eliminated relatively quickly from the rabbit VH with a first-order rate of vitreal elimination (k_1_) of 0.094 h^−1^, which corresponds to a half-life of 7.4 h. Drug concentration extrapolated at t = 0 was 2250 µg/mL, leading to a vitreal volume of distribution of 1.18 mL essentially identical to the vitreous volume. Based on these data, the calculated vitreal clearance was 0.11 mL/h. The data are summarized in Table 1.

**Table 1:** Pharmacokinetic parameters of THR-687 in rabbit and minipig

Parameter	Rabbit	Minipig
Vitreous volume (mL)	1.15	3.0
k_1_ (h^−1^)	0.094 [0.090–0.097]	0.056^a^
Vitreal half-life (h)	7.4 [7.1–7.7]	12.4
V_D,VH_ (mL)	1.18 [1.00–1.35]	–
CL_VH_ (mL/min)	0.110 [0.097–0.123]	–
k_2_ (h^−1^)	0.13 [0.12–0.14]	0.071 [0.056–0.087]
k_3_ (h^−1^)	0.066 [0.038–0.094]	0.13 [0.06–0.20]
k_4_ (h^−1^)	0.49 [0.39–0.59]	0.38 [0.22–0.53]
k_5_ (h^−1^)	2.07 [1.80–2.34]	1.86 [1.45–2.28]
t_1/2-term_ (h)	1.5 [1.2–1.8]	2.0 [1.5–3.2]
V_D,syst_ (L/kg)^b^	~ 0.2 [0–1.4]	~ 1.0 [0–2.9]
V_D,syst_ (L/kg)^c^	0.194 [0.186–0.203]	0.51 [0.46–0.56]
CL_syst_ (mL/min/kg)^c^	6.7 [6.4–7.0]	15.9 [14.4–17.5]

**Fig. 3:**
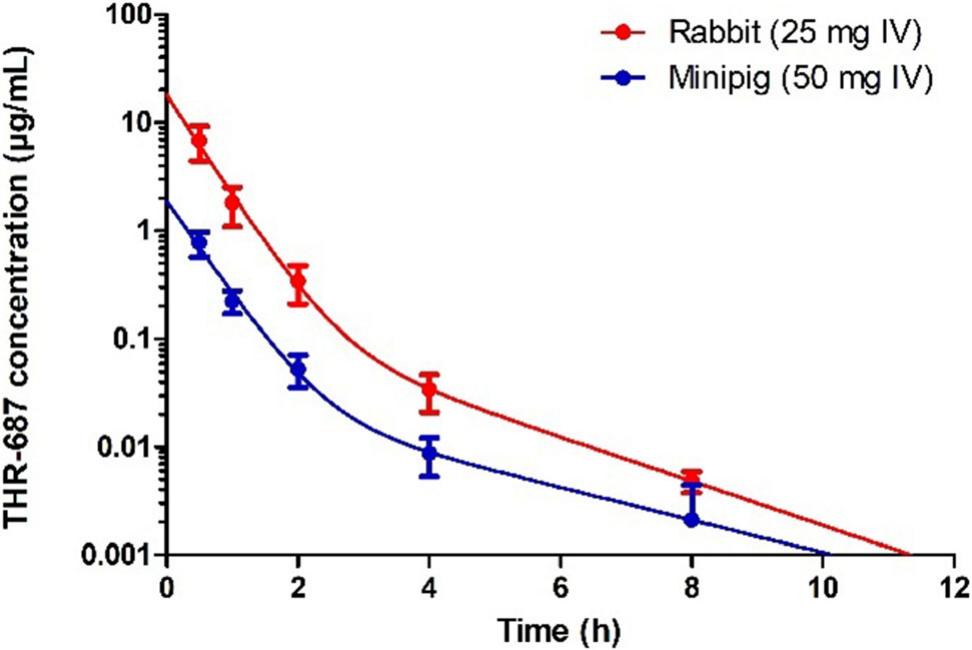
Drug plasma levels following intravenous administration of 25 mg and 50 mg of THR-687 in rabbit and minipig, respectively. Data are shown as mean ± SD. Data were analyzed based on a bi-compartmental model. The solid lines represent the best fit given by Eq. 4. Information extracted from these data is summarized in Table 1

The value of k_1_ for the rabbit was obtained from intravitreal pharmacokinetics (Fig. 2) and the one for the minipig ^a^ was calculated based on the eye size and geometry. The values of k_3_, k_4_, and k_5_ were obtained from intravenous pharmacokinetic experiments (Fig. 3). The values of k_2_ were calculated from plasma levels following IVT administration. ^b^Systemic volume of distribution from intravenous pharmacokinetic studies. ^c^Systemic volume of distribution and systemic clearance obtained from plasma levels following IVT administration

Intravitreal pharmacokinetics of THR-687 in the minipig was not determined experimentally, and we, therefore, chose to extrapolate this information from the rabbit data. We first obtained the vitreal clearance in the human eye (0.20 mL/h) using the empirical relationship between vitreal clearance in rabbit and human proposed by del Amo et al. [19], then calculated the predicted rate constant for vitreal elimination in human (0.044 h^−1^) as described in our accompanying paper. The same way as the systemic clearance represents the volume of plasma from which a substance is completely removed per unit time, the vitreal clearance can be interpreted as the volume of vitreous from which a drug is eliminated per unit time. Dividing the vitreal clearance by the vitreal volume of distribution leads to the rate constant for vitreal elimination (k_1_ = CL_VH_/V_D,VH_), a parameter that can thus be interpreted as the fraction of vitreous (or more exactly the fraction of the volume into which the drug distributes) from which the drug is removed per unit time. In the absence of a clear rationale to extrapolate pharmacokinetic properties from the rabbit or the human VH to the minipig VH, we assumed a linear relationship between k_1_ and the ratio between the surface of the eye (taken as a sphere) and its volume, i.e. 3/R (with R the radius of the eye). Calculating the geometry of the eye assuming vitreous volumes of 1.15, 3.0, and 4.36 mL for the rabbit, minipig, and human eye, respectively, allowed us to estimate a rate constant of vitreal elimination of 0.056 h^−1^ for THR-687 in the minipig.

### Intravenous pharmacokinetics

Plasma elimination following intravenous administration of THR-687 was evaluated in both rabbits and minipigs. In both species, the decrease in the circulating concentration of THR-687 was best described by the sum of two exponentials (Fig. 3). Data were thus analyzed based on a standard two-compartmental model (model (c) in Fig. 1) using Eq. 4 which allowed the determination of the first-order rate constants for transfer of the drug between the central and peripheral systemic compartments (k_3_ & k_4_), the rate constant for systemic elimination (k_5_), as well as the central systemic volume of distribution (V_D,syst_) (data summarized in Table 1). It should be noted, however, that, even though the value of all the individual rate constants characterizing the model (k_3_, k_4_, k_5_) could be obtained with a good precision for both species as demonstrated by the narrow 95% confidence interval on each of these parameters, the precision on the value of the central systemic volume of distribution was, because of the relatively large inter-animal variability and the very fast initial decrease in the drug circulating concentration, rather poor (Table 1) and should thus be regarded at this point as a rough approximation. The terminal half-life was similar in both species (Table 1).

### Plasma levels following intravitreal administration

**Fig. 4:**
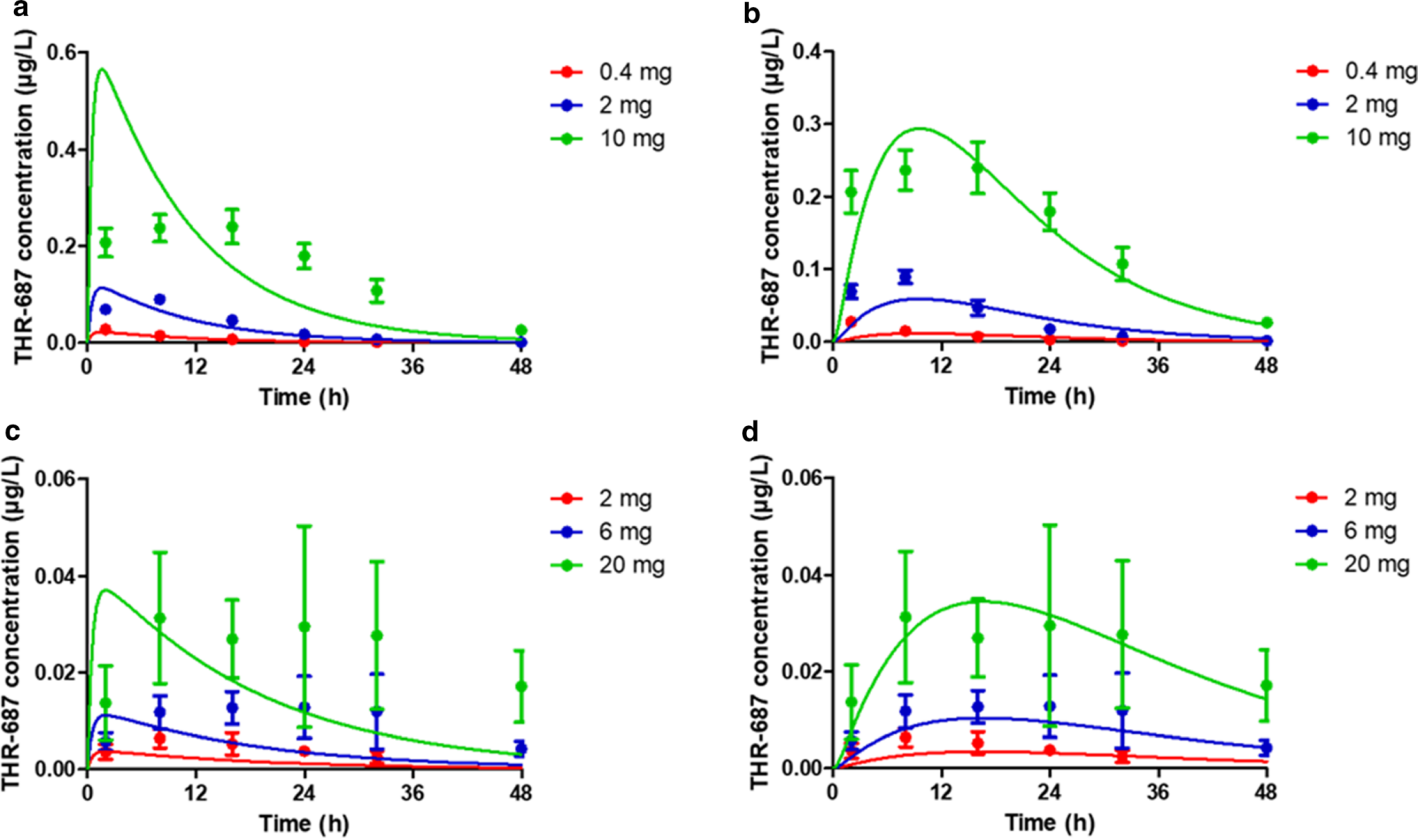
Plasma levels following bilateral IVT administration of THR-687 in rabbit (**a**, **b**) and minipig (**c**, **d**). Data are shown as mean ± SD. Doses indicated in the graph legends are total doses, i.e. twice the dose administered in each eye. The solid lines in graphs from panels a & c were generated from Eq. 12 using values for the individual rate constants (k_1_, k_3_, k_4_, and k_5_) as reported in Table 1 and values of 0.2 L/kg and 1.0 L/kg for the central systemic volume of distribution (V_D,syst_) in the rabbit (**a**) and minipig (**c**), respectively. The solid lines in graphs from panels b & d represent the best fit given by Eq. 19 using fixed values for the individual rate constants k_1_, k_3_, k_4_, and k_5_ as reported in Table 1, thus leaving k_2_ and V_D,syst_ as the only adjustable parameters

Systemic exposure (i.e. plasma levels over time) following IVT administration of THR-687 was evaluated in both rabbits and minipigs. Rabbits received bilateral IVT injections of 0.2, 1, and 5 mg THR-687 per eye and minipigs received bilateral IVT injections of 1, 3, and 10 mg THR-687 per eye. In both species, the administered drug appeared quickly in the circulation, being detected at the first time point (2 h). Drug levels then reached a flat peak roughly between 12 and 24 h post-injection in the rabbit and roughly 24 h post-injection in the minipig before decaying slowly (Fig. 4). Systemic exposure, expressed as the area under the curve (AUC) between 0 and 48 h determined by the trapezoidal method, was found to be directly proportional to the dose (not shown).

In our accompanying paper, we attempted to analyze circulating levels of the plasma kallikrein inhibitor THR-149 following IVT administration in rabbit based on the model reported by Xu et al. [20] and Zhang et al. [21] which assumes first-order absorption into and first-order elimination from the systemic circulation (model (e) in Fig. 1). Here, however, the latter model needs to be modified in order to incorporate the fact that THR-687 intravenous administration obeys bi-compartmental pharmacokinetics, thus becoming model (b) in Fig. 1. We showed in the Methods section that drug plasma levels for this model can be analyzed using Eq. 12 (elimination from each eye being assumed to be identical and additive). Since all rate constants describing the model (namely k_1_, k_3_, k_4_, and k_5_) in Eq. 12 also appear in Eq. 1 or Eq. 4, the value of all the parameters of Eq. 12 (including V_D,syst_) are known from either intravitreal or intravenous pharmacokinetics. Equation 12 should thus accurately predict drug circulating levels following IVT administration. Panels a & c of Fig. 4, however, show that this is not the case. In particular, and exactly as observed for THR-149, Eq. 12 predicts a maximum circulating drug concentration (C_max_) at ~ 1.6 h in the rabbit and ~ 2 h in the minipig while the observed C_max_ is very clearly reached significantly later.

Following the same reasoning as the one applied for THR-149 in our accompanying paper, we, therefore, asked whether the data could be more accurately interpreted using a four-compartment pharmacokinetic model that assumes the presence of an extra compartment that we will refer to here as the “ocular tissues compartment” through which the drug transits when being eliminated from the vitreous into the systemic compartment (model (a) in Fig. 1). Worth noting, and as discussed in our accompanying paper, the model only provides information on the drug distribution between the different compartments over time, but we cannot at this point speculate on which tissues or structures physically represent the ocular tissues compartment.

As shown in the Methods section, circulating drug levels for the latter model are represented by Eq. 19. Here too, information obtained from intravitreal and intravenous pharmacokinetic studies can be used to “educate” the model by attributing a fixed value to the parameters V_D,syst_, k_1_, k_3_, k_4_, and k_5_, thus leaving the sole k_2_ as a variable parameter. Alternatively—and this is what we chose to do given that the value of the central systemic volume of distribution was only poorly predicted from the intravenous pharmacokinetic studies—it is possible to attribute a fixed value only to the rate constants k_1_, k_3_, k_4_, and k_5_, thus allowing the determination of both V_D,syst_ and k_2_.

This analysis is shown in panels b & d of Fig. 4 for the rabbit and the minipig, respectively. Exactly as observed for THR-149, interpretation the data based on a model that assumes the existence of an “ocular tissues” compartment allowed a much more accurate prediction of the experimental data. Both k_2_ and V_D,syst_ could be extracted with good precision (Table 1), the value of V_D,syst_ remaining well within the range predicted by intravenous studies. This allowed us to calculate the systemic clearance in these two species (Eq. 5). These values are reported in Table 1. Importantly, since all rate constants except k_2_ were attributed their respective values as determined from separate intravitreal and intravenous pharmacokinetic studies, all data sets remain perfectly coherent.

**Fig. 5:**
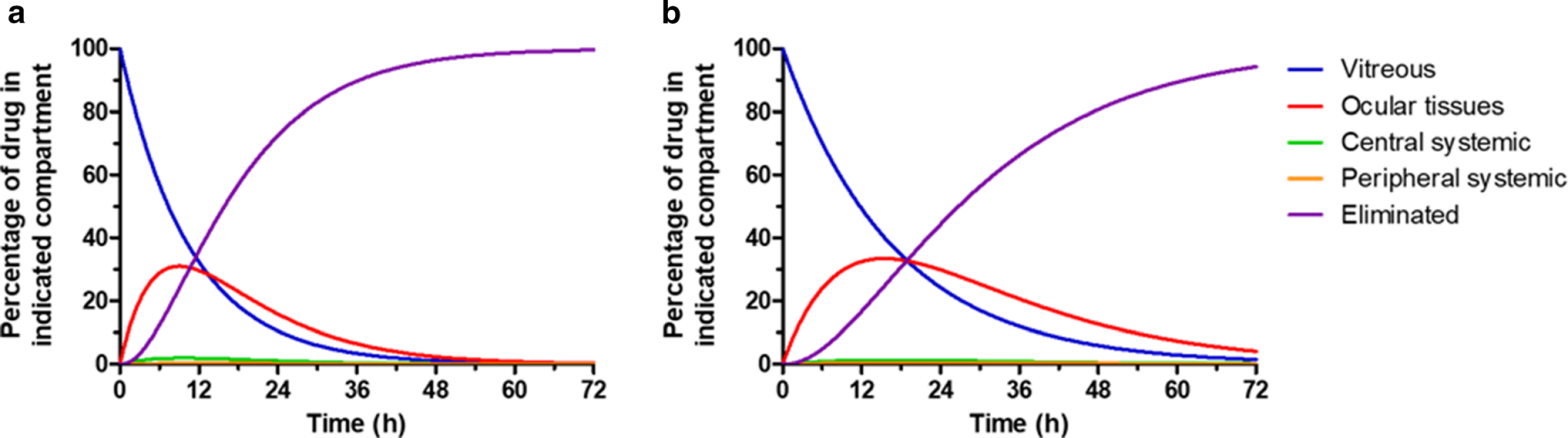
Percentage of drug present in each of the compartments (VH, ocular tissues, central systemic, and peripheral systemic) and total percentage of drug eliminated following IVT administration of THR-687 in rabbit (**a**) and minipig (**b**), as predicted based on pharmacokinetic model (**a**) from Fig. 1. Calculations were based on Eq. 13–17 assuming values for the individual rate constants (k_1_, k_2_, k_3_, k_4_, and k_5_) and for the central systemic volume of distribution (V_D,syst_) as reported in Table 1

Finally, as already reported in our accompanying paper, Eq. 13–17 allow to calculate the percentage of drug present in each of the compartments of the model (i.e. VH, ocular tissues, central systemic, and peripheral systemic) as well as the percentage of drug eliminated by the organism at any moment in time. These simulations, represented in Fig. 5, predict that the fraction of drug present in the ocular tissues compartment will be highest ~ 9 h (rabbit) and 15–16 h (minipig) post-administration, reaching ~ 30–35% of the total administered dose in both species. By contrast, because systemic elimination is faster than ocular elimination in both species, the percentage of drug present in the systemic compartments is predicted to remain below 2% of the total administered drug at any point in time.

### Binding to plasma proteins and relationship with systemic volume of distribution

THR-687 exhibits a central systemic volume of distribution (V_D,syst_) that is somewhat different in the rabbit and in the minipig (~ 0.2 L/kg in the rabbit vs. ~ 0.5 L/kg in the minipig). This could be explained by species differences in the binding of the drug to plasma proteins [22]. We, therefore, assessed the extent of protein binding in rabbit, pig, and also human plasma using a conventional equilibrium dialysis assay, and found that protein binding, although significant in plasma from all three species, is identical (within experimental variability) in human and rabbit but lower in pig plasma (percentage of bound drug being 92 ± 3%, 78 ± 7%, and 91 ± 3%—mean ± SD—in rabbit, pig, and human plasma, respectively). Noteworthy, the apparent volume of distribution calculated based on the free (i.e. unbound) drug concentration in plasma is similar (~ 2.3–2.4 L/kg) in the rabbit and the minipig. The data reported here also allow to predict that V_D,syst_ in human is likely to be close to the one observed in the rabbit, i.e. 0.2 L/kg.

### Systemic exposure following intravenous and intravitreal administration

Systemic exposure, whether following IVT or intravenous drug administration, can be represented by the total area under the curve (AUC_0-∞_) for the graph of plasma concentration *vs*. time. This value can be determined by integrating Eq. 4 or Eq. 19 between 0 and ∞ (Eq. 20 and 21) which is straightforward for a sum of exponentials (see Eq. 14 in our accompanying paper).

20 $${\int }_{0}^{\infty }Eq. 18= \frac{D\cdot {k}_{1}\cdot {k}_{2}}{{V}_{D,syst}\cdot \left({k}_{1}-{k}_{2}\right)\cdot M\cdot R\cdot N}\cdot \left\{\frac{\left({k}_{1}-{k}_{4}\right)\cdot R\cdot N}{{k}_{1}}-\frac{\left({k}_{2}-{k}_{4}\right)\cdot M\cdot N}{{k}_{2}}+\frac{-\left({k}_{1}-{k}_{4}\right)\cdot R\cdot N+\left({k}_{2}-{k}_{4}\right)\cdot M\cdot N+U}{\alpha }-\frac{U}{\beta }\right\}$$

21 $${\int }_{0}^{\infty }Eq. 14=\frac{D}{{V}_{D,syst}\cdot \left(\beta -\alpha \right)}\cdot \left(\frac{{k}_{4}-\alpha }{\alpha }+\frac{\beta -{k}_{4}}{\beta }\right)=\frac{D}{{V}_{D,syst}\cdot {k}_{5}}=\frac{D}{{CL}_{syst}}$$

Equation 20 & 21 are identical and return exactly the same value for a given set of constants, indicating that systemic exposure is independent of the route of administration or, in the case of IVT administration, of the rate of ocular elimination, and depends only on the dose, the central systemic volume of distribution, and the rate of elimination from the central systemic compartment or, alternatively, on the dose and the systemic clearance. Noteworthy, we reported the same finding for model (d) of Fig. 1 in our accompanying paper.

### Extrapolation to human

**Fig. 6:**
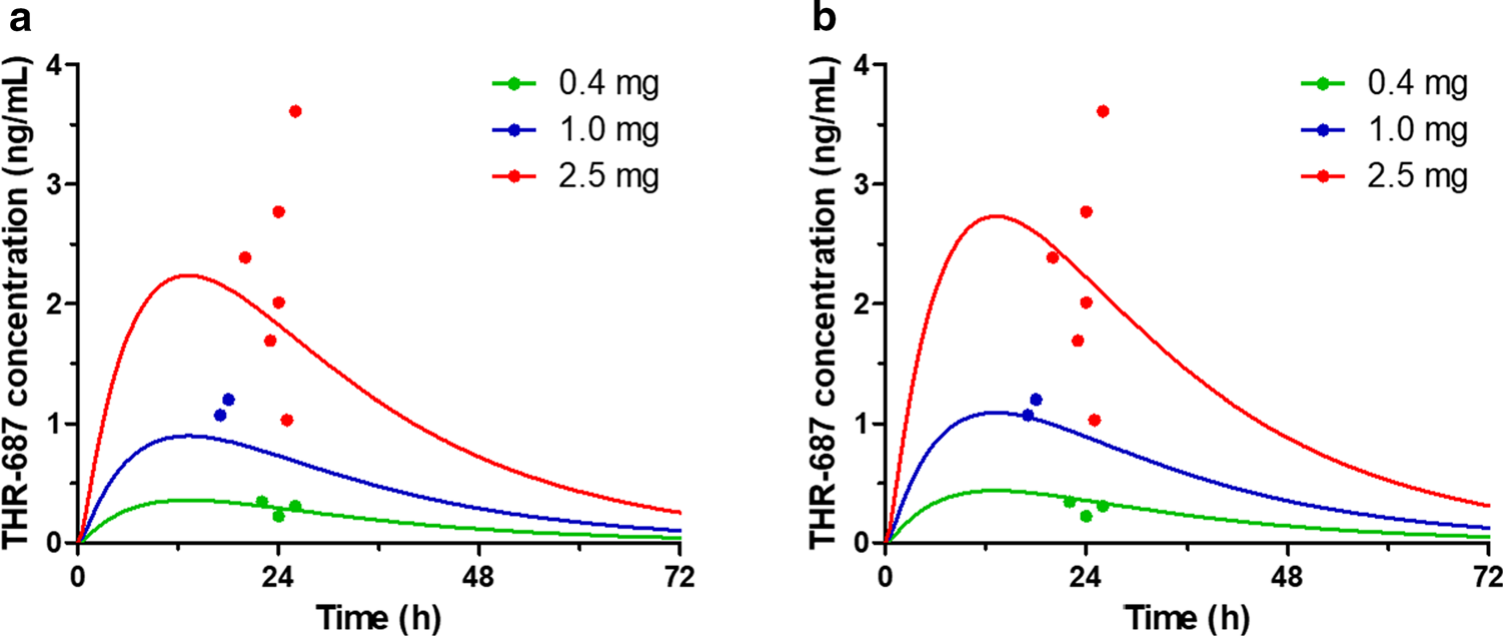
Plasma levels following single IVT administration of the indicated dose of THR-687 in human as predicted by pharmacokinetic model (**a**) from Fig. 1 using Eq. 19. **a** Parameters used for the calculations were as follows: body weight = 70 kg; central systemic volume of distribution (V_D,syst_) = 13.6 L (0.194 L/kg); k_1_ = 0.044 h^−1^; k_2_ = 0.13 h^−1^; k_3_ = 0.066 h^−1^; k_4_ = 0.49 h^−1^; and k_5_ = 2.07 h^−1^. **b** The solid lines represent the best fit given by Eq. 19 with V_D,syst_ as the sole adjustable parameter, the values of the individual rate constants being maintained to those used in (**a**). The dots represent plasma levels measured in patients during Ph I evaluation of the drug

Pharmacokinetic data in preclinical models can also be used to extrapolate or predict pharmacokinetics in human. The vitreal clearance (CL_VH_) in the human eye can easily be extrapolated from the vitreal clearance in the rabbit ([19]—see also our accompanying paper). From this, and extrapolating the vitreal volume of distribution of the human eye as described in our accompanying paper, one can predict a value of 0.044 h^−1^ for the rate constant of elimination of THR-687 in the human VH (Eq. 3), which corresponds to a half-life of 16 h. The value of rate constants describing systemic distribution and elimination of THR-687 (i.e. k_3_, k_4_, and k_5_) are very close in the rabbit and the minipig despite the difference in size between these two species, which suggests that systemic pharmacokinetics in human will be accurately described using similar values for the rate constants. However, the central systemic volume of distribution (V_D,syst_) is more likely to resemble the one measured in rabbit, and we, therefore, chose to attribute to rate constants k_3_, k_4_, and k_5_ in human the same values as those measured in the rabbit. Finally, in the absence of a clear rationale to extrapolate the value of k_2_ in human, we also opted to attribute for pharmacokinetic simulations in human a value of k_2_ identical to the one determined in rabbit. Based on the above numbers, it is thus possible, using Eq. 19, to predict THR-687 circulating levels in human following IVT administration. These findings are presented in Fig. 6a for the 3 doses tested in the clinic during Ph I evaluation of THR-687 in DME patients, namely 2.5 mg, 1.0 mg, and 0.4 mg per eye (monolateral). As can be seen, these predictions are in excellent agreement with plasma levels recorded roughly 24 h post-administration during the trial. Analyzing the data with Eq. 19 with V_D,syst_ as the only fitting parameter only resulted in a minimal optimization of the value of V_D,syst_ in human (0.16 vs. 0.19 L/kg) (Fig. 6b). THR-687 plasma concentration is expected to reach a maximum value of only 2.7 ng/mL for the highest dose of 2.5 mg/eye roughly 13 h post-administration before decaying slowly. Predicted systemic exposure is calculated to be 108 (ng/mL).h for the highest dose based on Eq. 20.

## Discussion

THR-687 is a relatively hydrophilic (logP = -3) small molecule drug. Following IVT administration in rabbit, THR-687 is eliminated relatively quickly from the vitreous with a half-life of 7.4 h. There are two major routes of drug elimination from the vitreous, i.e. anterior and posterior. Drugs eliminated via the anterior route diffuse across the lens and the ciliary body to the posterior chamber, then reach the anterior chamber through the aqueous humor turnover to be finally eliminated by the trabecular and uveoscleral outflow. The anterior route is accessible to all types of drugs but is typically predominant for small hydrophilic molecules or large molecules that are not able to cross the retina. Posterior elimination implies permeation through the retina and subsequent elimination by the choroidal blood flow. Choroidal elimination is fast because blood flow is high and choroidal capillaries are highly-fenestrated, and the diffusion of drugs through the retina is thus predominantly from inner to outer. The posterior route is the main elimination pathway for small and lipophilic molecules [4, 5, 19]. With a value of 0.11 mL/h, the vitreal clearance of THR-687 in the rabbit eye is well within the vitreal clearance range observed for small hydrophilic drugs and significantly lower than the average vitreal clearance of small lipophilic molecules [5, 19]. Together with the general physico-chemical properties of the molecule, this suggests that vitreal elimination of THR-687 occurs primarily via the anterior route with limited elimination through the posterior route and the blood-retinal barrier.

Systemic elimination following intravenous administration in either the rabbit or the minipig was best represented by a bi-compartmental pharmacokinetic model. Interestingly, despite the difference in size between these two species, the rate constants describing the model were very similar in the rabbit and the minipig, suggesting that these constants can be extrapolated to human. The apparent central systemic volume of distribution was found to be relatively small in both species, close to extracellular fluid in the rabbit (~ 0.2 L/kg) and to total body water in the minipig (~ 0.5 L/kg). THR-687 shows extensive binding to plasma proteins, and differences in the extent of protein binding in rabbit and pig plasma adequately explains the apparent discrepancy in the volume of distribution between the two species [22]. Systemic clearance is close to glomerular filtration rate in the rabbit [23] but significantly higher in the minipig. Here too, though, these differences can be explained by variations in the extent of plasma protein binding between the two species since it is the volume of distribution rather than the rate constant for drug elimination from the central systemic compartment that differs between the two species (Eq. 5). By contrast, the terminal half-life was similar in both species (1.5 and 2.0 h in rabbit and minipig, respectively).

Circulating drug levels following IVT administration in rabbit or minipig were well described by a model which assumes the existence of an additional compartment localized between the VH compartment itself (i.e. the compartment where the drug is administered) and the central systemic compartment (model (a) from Fig. 1). This pharmacokinetic model is similar to the one we used to describe plasma levels of the plasma kallikrein inhibitor THR-149 following eye administration (our accompanying paper, also model (d) from Fig. 1), except that the model applied to THR-687 accounts for bi-compartmental systemic distribution. In this respect, model (a) from Fig. 1 can be regarded as the most general and broadly applicable of the models discussed here. Of note, numerical simulations show, as also illustrated in Fig. 1, that the equations that apply to this model (most notably Eq. 19) simplify into those that describe less complex models when e.g. k_2_ is very large (which eliminates the ocular tissues compartment), when both k_2_ and k_1_ are very large (which is equivalent to performing an IV administration), or when k_3_ is equal to zero (which eliminates the peripheral systemic compartment). For example, in practice, it is possible that data analysis using Eq. 19 would return a value of k_2_ much larger than the one of k_1_, which would not invalidate the physical existence of the ocular tissues compartment but simply indicate that the studied drug does accumulate significantly in said compartment.

Finally, the prediction of THR-687 circulating levels following intravitreal administration in human was in remarkably good agreement with available data, highlighting the relevance of the proposed model for the evaluation of systemic exposure following intravitreal administration in a clinical setting.

## Conclusion

We show here and in our accompanying paper that circulating levels following IVT administration of two structurally very distinct drugs can be accurately represented on the basis of models that assume the existence of an ocular tissues compartment localized between the VH compartment itself and the systemic compartment. This suggests that these models will also accurately describe circulating levels of a majority of drugs administered in the eye. We also provide an analytical solution to the systems of linear differential equations that describe the proposed models, thereby eliminating the need for complex software’s capable of handling differential equations and allowing data treatment with standard nonlinear regression analysis.

## Appendix

Solution/integration of Eq. syst. 1 based upon the methodology described in our accompanying paper.

$$ \begin{aligned} dA/dt & = - k_{1} \cdot A\quad {\text{Eq}}.{\text{syst}}{.1} \\ dB/dt & = k_{1} \cdot A - (k_{3} + k_{5} ) \cdot B + k_{4} \cdot C \\ dC/dt & = k_{3} \cdot B - k_{4} \cdot C \\ dD/dt & = k_{5} \cdot B \\ \end{aligned} $$

Primary system matrix.

$$S=\left(\begin{array}{c}-{k}_{1}\\ {k}_{1}\\ 0\\ 0\end{array} \begin{array}{c}0\\ -\left({k}_{3}+{k}_{5}\right)\\ {k}_{3}\\ {k}_{5}\end{array} \begin{array}{c}0\\ {k}_{4}\\ {-k}_{4}\\ 0\end{array} \begin{array}{c}0\\ 0\\ 0\\ 0\end{array}\right)$$

Eigenvalues.

$$ \begin{aligned} \lambda_{1} & = - k_{1} \\ \lambda_{2} & = - \frac{1}{2} \cdot \left\{ {k_{3} + k_{4} + k_{5} + \sqrt {\left( {k_{3} + k_{4} + k_{5} } \right)^{2} - 4 \cdot k_{4} \cdot k_{5} } } \right\} = - \alpha \\ \lambda_{3} & = - \frac{1}{2} \cdot \left\{ {k_{3} + k_{4} + k_{5} - \sqrt {\left( {k_{3} + k_{4} + k_{5} } \right)^{2} - 4 \cdot k_{4} \cdot k_{5} } } \right\} = - \beta \\ \lambda_{4} & = 0 \\ \end{aligned} $$

Squared matrix P.

$$P=\left(\begin{array}{c}\left({k}_{1}/{k}_{5}\right)\cdot \left(1-{k}_{3}/\left({k}_{1}-{k}_{4}\right)\right)-1\\ -\left({k}_{1}/{k}_{5}\right)\\ \left({k}_{1}{k}_{3}\right)/\left({k}_{5}\left({k}_{1}-{k}_{4}\right)\right)\\ 1\end{array} \begin{array}{c}0\\ -\left(\alpha /{k}_{5}\right)\\ \left(\alpha /{k}_{5}\right)-1\\ 1\end{array} \begin{array}{c}0\\ -\left(\beta /{k}_{5}\right)\\ \left(\beta /{k}_{5}\right)-1\\ 1\end{array} \begin{array}{c}0\\ 0\\ 0\\ 1\end{array}\right)$$

Expression of C_1_, C_2_, C_3_, and C_4_.

$$ \begin{aligned} C_{1} & = A_{0} \cdot \frac{{k_{5} \cdot \left( {k_{1} - k_{4} } \right)}}{{\left( {k_{1} - \alpha } \right) \cdot \left( {k_{1} - \beta } \right)}} \\ C_{2} & = A_{0} \cdot \frac{{k_{1} \cdot \left[ {k_{3} \cdot \beta - \left( {\beta - k_{5} } \right) \cdot \left( {k_{1} - k_{4} } \right)} \right]}}{{\left( {\beta - \alpha } \right)\left( {k_{1} - \alpha } \right) \cdot \left( {k_{1} - \beta } \right)}} \\ C_{3} & = A_{0} \cdot \frac{{k_{1} \cdot \left[ {\left( {\alpha - k_{5} } \right) \cdot \left( {k_{1} - k_{4} } \right) - k_{3} \cdot \alpha } \right]}}{{\left( {\beta - \alpha } \right)\left( {k_{1} - \alpha } \right) \cdot \left( {k_{1} - \beta } \right)}} \\ C_{4} & = A_{0} \cdot \frac{{k_{5} \cdot \left( {k_{1} - k_{4} } \right) - k_{1} \cdot \left( {k_{1} - k_{3} - k_{4} } \right)}}{{\left( {k_{1} - \alpha } \right) \cdot \left( {\beta - k_{1} } \right)}} \\ \end{aligned} $$

with

$$ \begin{aligned} \alpha & = \left( {1/2} \right) \cdot \left\{ {k_{3} + k_{4} + k_{5} + \sqrt {\left( {k_{3} + k_{4} + k_{5} } \right)^{2} - 4 \cdot k_{4} \cdot k_{5} } } \right\} \\ \beta & = \left( {1/2} \right) \cdot \left\{ {k_{3} + k_{4} + k_{5} - \sqrt {\left( {k_{3} + k_{4} + k_{5} } \right)^{2} - 4 \cdot k_{4} \cdot k_{5} } } \right\} \\ \end{aligned} $$

Solution/integration of Eq. syst. 2.

$$ \begin{aligned} dA/dt & = - k_{1} \cdot A\quad {\text{Eq}}.{\text{syst}}{.2} \\ db/dt & = k_{1} \cdot A - k_{2} \cdot B \\ dc/dt & = k_{2} \cdot B - (k_{3} + k_{5} ) \cdot C + k_{4} \cdot D \\ dD/dt & = k_{3} \cdot C - k_{4} \cdot D \\ dE/dt & = k_{5} \cdot C \\ \end{aligned} $$

Primary system matrix.

$$S=\left(\begin{array}{c}-{k}_{1}\\ {k}_{1}\\ 0\\ 0\\ 0\end{array} \begin{array}{c}0\\ -{k}_{2}\\ {k}_{2}\\ 0\\ 0\end{array} \begin{array}{c}0\\ 0\\ -\left({{k}_{3}+k}_{5}\right)\\ {k}_{3}\\ {k}_{5}\end{array} \begin{array}{c}0\\ 0\\ {k}_{4}\\ {-k}_{4}\\ 0\end{array} \begin{array}{c}0\\ 0\\ 0\\ 0\\ 0\end{array}\right)$$

Eigenvalues.

$$ \begin{aligned} \lambda_{1} & = - k_{1} \\ \lambda_{2} & = - k_{2} \\ \lambda_{3} & = - \frac{1}{2} \cdot \left\{ {k_{3} + k_{4} + k_{5} + \sqrt {\left( {k_{3} + k_{4} + k_{5} } \right)^{2} - 4 \cdot k_{4} \cdot k_{5} } } \right\} = - \alpha \\ \lambda_{4} & = - \frac{1}{2} \cdot \left\{ {k_{3} + k_{4} + k_{5} - \sqrt {\left( {k_{3} + k_{4} + k_{5} } \right)^{2} - 4 \cdot k_{4}\cdot k_{5} } } \right\} = - \beta \\ \lambda_{5} & = 0 \\ \end{aligned} $$

Squared matrix P.

$$P=\left(\begin{array}{c}-\left(\left({k}_{1}-{k}_{2}\right)M\right)/\left({{k}_{2}k}_{5}\left({k}_{1}-{k}_{4}\right)\right)\\ \left({k}_{1}M\right)/\left({{k}_{2}k}_{5}\left({k}_{1}-{k}_{4}\right)\right)\\ -\left({k}_{1}/{k}_{5}\right)\\ \left({k}_{1}{k}_{3}\right)/\left({k}_{5}\left({k}_{1}-{k}_{4}\right)\right)\\ 1\end{array} \begin{array}{c}0\\ -\left({k}_{3}{k}_{4}\right)/\left({k}_{5}\left({k}_{2}-{k}_{4}\right)\right)-\left({-k}_{2}+{k}_{3}+{k}_{5}\right)/\left({k}_{5}\right)\\ -\left({k}_{2}/{k}_{5}\right)\\ \left({k}_{2}{k}_{3}\right)/\left({k}_{5}\left({k}_{2}-{k}_{4}\right)\right)\\ 1\end{array} \begin{array}{c}0\\ 0\\ -\left(\alpha /{k}_{5}\right)\\ \left({k}_{3}\alpha \right)/\left({k}_{5}\left(\alpha -{k}_{4}\right)\right)\\ 1\end{array} \begin{array}{c}0\\ 0\\ -\left(\beta /{k}_{5}\right)\\ \left({k}_{3}\beta \right)/\left({k}_{5}\left(\beta -{k}_{4}\right)\right)\\ 1\end{array} \begin{array}{c}0\\ 0\\ 0\\ 0\\ 1\end{array}\right)$$

Expression of C_1_, C_2_, C_3_, C_4_, and C_5_.

$$ \begin{aligned} C_{1} & = - A_{0} \cdot \frac{{k_{2} \cdot k_{5} \cdot \left( {k_{1} - k_{4} } \right)}}{{\left( {k_{1} - k_{2} } \right) \cdot M}} \\ C_{2} & = A_{0} \cdot \frac{{k_{1} \cdot k_{5} \cdot \left( {k_{2} - k_{4} } \right)}}{{\left( {k_{1} - k_{2} } \right) \cdot R}} \\ C_{3} & = - A_{0} \cdot \frac{{k_{1} \cdot k_{2} \cdot k_{5} \cdot \left[ { - \left( {k_{1} - k_{4} } \right) \cdot R \cdot N + \left( {k_{2} - k_{4} } \right) \cdot M \cdot N + U} \right]}}{{\alpha \cdot M \cdot R \cdot N \cdot \left( {k_{1} - k_{2} } \right)}} \\ C_{4} & = A_{0} \cdot \frac{{k_{1} \cdot k_{2} \cdot k_{5} \cdot U}}{{\beta \cdot M \cdot R \cdot N \cdot \left( {k_{1} - k_{2} } \right)}} \\ C_{5} & = A_{0} \cdot \frac{{k_{5} \cdot \left\{ {k_{2} \cdot \left( {k_{1} - k_{4} } \right) \cdot \alpha \cdot \beta \cdot R \cdot N - k_{1} \cdot \left( {k_{2} - k_{4} } \right) \cdot \alpha \cdot \beta \cdot M \cdot N + k_{1} \cdot k_{2} \cdot \beta \cdot \left[ { - \left( {k_{1} - k_{4} } \right) \cdot R \cdot N + \left( {k_{2} - k_{4} } \right) \cdot M \cdot N + U} \right] - k_{1} \cdot k_{2} \cdot \alpha \cdot U} \right\}}}{{\alpha \cdot \beta \cdot M \cdot R \cdot N \cdot \left( {k_{1} - k_{2} } \right)}} \\ \end{aligned} $$

with

$$ \begin{aligned} \alpha & = \left( {1/2} \right) \cdot \left\{ {k_{3} + k_{4} + k_{5} + \sqrt {\left( {k_{3} + k_{4} + k_{5} } \right)^{2} - 4 \cdot k_{4} \cdot k_{5} } } \right\} \\ \beta & = \left( {1/2} \right) \cdot \left\{ {k_{3} + k_{4} + k_{5} - \sqrt {\left( {k_{3} + k_{4} + k_{5} } \right)^{2} - 4 \cdot k_{4} \cdot k_{5} } } \right\} \\ M & = k_{1}^{2} - k_{1} \cdot k_{3} - k_{1} \cdot k_{4} - k_{1} \cdot k_{5} + k_{4} \cdot k_{5} \\ R & = k_{2}^{2} - k_{2} \cdot k_{3} - k_{2} \cdot k_{4} - k_{2} \cdot k_{5} + k_{4} \cdot k_{5} \\ N & = \sqrt {\left( {k_{3} + k_{4} + k_{5} } \right)^{2} - 4 \cdot k_{4} \cdot k_{5} } \\ U & = \left( {\beta - k_{4} } \right) \cdot \left\{ {R \cdot \left( {\alpha - k_{1} } \right) - M \cdot \left( {\alpha - k_{2} } \right)} \right\} \\ \end{aligned} $$

## References

[1] Kowluru A, Zhong Q (2011). Beyond AREDS: is there a place for antioxidant therapy in the prevention/treatment of eye disease?. Invest Ophth Vis Sci.

[2] Bourne RR, Stevens GA, White RA, Smith JL, Flaxman SR, Price H, Jonas JB, Keeffe J, Leasher J, Naidoo K, Pesudovs K, Resniko S, Taylor HR (2013). Causes of vision loss worldwide, 1990–2010: a systematic analysis. Lancet Glob Health.

[3] Wang W, Wang F, Qin W, Liu H, Lu B, Chung C, Zhu J, Gu Q, Shi W, Wen C, Wu F, Zhang K, Sun X (2016). Joint antiangiogenic effect of ATN-161 and anti-VEGF antibody in a rat model of early wet age-related macular degeneration. Mol Pharm.

[4] Varela-Fernández R, Díaz-Tomé V, Luaces-Rodríguez A, Conde-Penedo A, García-Otero X, Luzardo-Álvarez A, Fernández-Ferreiro A, Otero-Espinar FJ (2020). Drug delivery to the posterior segment of the eye: biopharmaceutic and pharmacokinetic considerations. Pharmaceutics.

[5] del Amo EM, Rimpelä A-K, Heikkinen E, Kari OK, Ramsay E, Lajunen T, Schmitt M, Pelkonen L, Bhattacharya M, Richardson D, Subrizi A, Turunen T, Reinisalo M, Itkonen J, Toropainen E, Casteleijn M, Kidron H, Antopolsky M, Vellonen K-S, Ruponen M, Urtti A (2017). Pharmacokinetic aspects of retinal drug delivery. Prog Retin Eye Res.

[6] Deng W, Liu C, Parra C, Sims JR, Faiq MA, Sainulabdeen A, Song H, Chan KC (2020). Quantitative imaging of the clearance systems in the eye and the brain. Quant Imaging Med Surg.

[7] Wang X, Lou N, Eberhardt A, Yang Y, Kusk P, Xu Q, Förstera B, Peng S, Shi M, Ladrón-de-Guevara A, Delle C, Sigurdsson B, Xavier ALR, Ertürk A, Libby RT, Chen L, Thrane AS, Nedergaard M (2020). An ocular glymphatic clearance system removes β-amyloid from the rodent eye. Sci Transl Med.

[8] Yucel Y, Gupta N (2015). Lymphatic drainage from the eye: a new target for therapy. Prog Brain Res.

[9] Dias CS, Anand BS, Mitra AK (2002). Effect of mono- and di-acylation on the ocular disposition of ganciclovir: physicochemical properties, ocular bioreversion, and antiviral activity of short chain ester prodrugs. J Pharm Sci.

[10] Duvvuri S, Majumdar S, Mitra AK (2004). Role of metabolism in ocular drug delivery. Curr Drug Metab.

[11] Gurreri A, Pazzaglia A (2021). Diabetic macular edema: state of art and intraocular pharmacological approaches. Adv Exp Med Biol.

[12] Chakravarthy U, Harding SP, Rogers CA, Downes SM, Lotery AJ, Culliford LA, Reeves BC, IVAN Study Investigators (2013). Alternative treatments to inhibit VEGF in age-related choroidal neovascularisation: 2-year findings of the IVAN randomised controlled trial. Lancet.

[13] Csaky K, Do DV (2009). Safety implications of vascular endothelial growth factor blockade for subjects receiving intravitreal anti-vascular endothelial growth factor therapies. Am J Ophthalmol.

[14] Hirano T, Toriyama Y, Iesato Y, Imai A, Murata T (2018). Changes in plasma vascular endothelial growth factor level after intravitreal injection of bevacizumab, aflibercept, or ranibizumab for diabetic macular edema. Retina.

[15] Rogers CA, Scott LJ, Reeves BC, Downes S, Lotery AJ, Dick AD, Chakravarthy U (2018). Serum vascular endothelial growth factor levels in the IVAN trial; relationships with drug, dosing, and systemic serious adverse events. Ophthalmol Retina.

[16] Hu T-T, Vanhove M, Porcu M, Van Hove I, Van Bergen T, Jonckx B, Barbeaux P, Vermassen E, Feyen JHM (2019). The potent small molecule integrin antagonist THR-687 is a promising next generation therapy for retinal vascular disorders. Exp Eye Res.

[17] Hull CJ (1979). Pharmacokinetics and pharmacodynamics. Br J Anaesth.

[18] Del Amo EM, Urtti A (2015). Rabbit as an animal model for intravitreal pharmacokinetics: clinical predictability and quality of the published data. Exp Eye Res.

[19] del Amo EM, Vellonen K-S, Kidron H, Urtti A (2015). Intravitreal clearance and volume of distribution of compounds in rabbits: In silico prediction and pharmacokinetic simulations for drug development. Eur J Pharm Biopharm.

[20] Xu L, Lu T, Tuomi L, Jumbe N, Lu J, Eppler S, Kuebler P, Damico-Beyer LA, Joshi A (2013). Pharmacokinetics of ranibizumab in patients with neovascular age-related macular degeneration: a population approach. Invest Ophthalmol Vis Sci.

[21] Zhang Y, Yao Z, Kaila N, Kuebler P, Visich J, Maia M, Tuomi L, Ehrlich JS, Rubio RG, Campochiaro PA (2014). Pharmacokinetics of ranibizumab after intravitreal administration in patients with retinal vein occlusion or diabetic macular edema. Ophthalmology.

[22] Holford N, Yim D-S (2016) Volume of distribution. Transl Clin Pharmacol 24:74-77

[23] Lippi I, Perondi F, Petrini D, La Fortuna MC, Luci G, Intorre L, Guidi G, Meucci V (2019). Evaluation of glomerular filtration rate estimation by means of plasma clearance of iohexol in domestic rabbits (*Oryctolagus cuniculus*). Am J Vet Res.

